# A standard numbering scheme for thiamine diphosphate-dependent decarboxylases

**DOI:** 10.1186/1471-2091-13-24

**Published:** 2012-11-17

**Authors:** Constantin Vogel, Michael Widmann, Martina Pohl, Jürgen Pleiss

**Affiliations:** 1 Institute of Technical Biochemistry, University of Stuttgart, Allmandring 31, Stuttgart, 70569, Germany; 2 IBG-1: Biotechnology, Forschungszentrum Jülich GmbH, Jülich, 52425, Germany

## Abstract

**Background:**

Standard numbering schemes for families of homologous proteins allow for the unambiguous identification of functionally and structurally relevant residues, to communicate results on mutations, and to systematically analyse sequence-function relationships in protein families. Standard numbering schemes have been successfully implemented for several protein families, including lactamases and antibodies, whereas a numbering scheme for the structural family of thiamine-diphosphate (ThDP) -dependent decarboxylases, a large subfamily of the class of ThDP-dependent enzymes encompassing pyruvate-, benzoylformate-, 2-oxo acid-, indolpyruvate- and phenylpyruvate decarboxylases, benzaldehyde lyase, acetohydroxyacid synthases and 2-succinyl-5-enolpyruvyl-6-hydroxy-3-cyclohexadiene-1-carboxylate synthase (MenD) is still missing.

Despite a high structural similarity between the members of the ThDP-dependent decarboxylases, their sequences are diverse and make a pairwise sequence comparison of protein family members difficult.

**Results:**

We developed and validated a standard numbering scheme for the family of ThDP-dependent decarboxylases. A profile hidden Markov model (HMM) was created using a set of representative sequences from the family of ThDP-dependent decarboxylases. The pyruvate decarboxylase from *S*. *cerevisiae* (PDB: 2VK8) was chosen as a reference because it is a well characterized enzyme. The crystal structure with the PDB identifier 2VK8 encompasses the structure of the *Sc*PDC mutant E477Q, the cofactors ThDP and Mg^2+^ as well as the substrate analogue (2S)-2-hydroxypropanoic acid. The absolute numbering of this reference sequence was transferred to all members of the ThDP-dependent decarboxylase protein family. Subsequently, the numbering scheme was integrated into the already established Thiamine-diphosphate dependent Enzyme Engineering Database (TEED) and was used to systematically analyze functionally and structurally relevant positions in the superfamily of ThDP-dependent decarboxylases.

**Conclusions:**

The numbering scheme serves as a tool for the reliable sequence alignment of ThDP-dependent decarboxylases and the unambiguous identification and communication of corresponding positions. Thus, it is the basis for the systematic and automated analysis of sequence-encoded properties such as structural and functional relevance of amino acid positions, because the analysis of conserved positions, the identification of correlated mutations and the determination of subfamily specific amino acid distributions depend on reliable multisequence alignments and the unambiguous identification of the alignment columns. The method is reliable and robust and can easily be adapted to further protein families.

## Background

Thiamine diphosphate (ThDP) -dependent decarboxylases are a large subfamily of the class of ThDP-dependent enzymes which are essential in many biosynthetic pathways. Due to the scientific and industrial relevance of enzymes capable of catalysing C-C bond formation and cleavage, we have focused in this work on the decarboxylase superfamily of the ThDP-dependent Enzyme Engineering Database (TEED) [1]. This superfamily contains among others pyruvate decarboxylases (PDCs, EC 4.1.1.1), indolepyruvate decarboxylases (IPDCs, EC 4.1.1.74), phenyl pyruvate oxidases (POXs, EC 1.2.3.3), the E1 component of pyruvate dehydrogenases (PDHs, EC 1.2.4.1), oxalyl-CoA decarboxylases (OCDCs, EC 4.1.1.8), benzaldehyde lyases (BALs, EC 4.1.2.38), benzoylformate decarboxylases (BFDs, EC 4.1.1.7), acetohydroxyacid synthases (AHASs, EC 2.2.1.6), glyoxylate carboligases (GXCs, EC 4.1.1.47), sulfoacetaldehyde acetyltransferases (SAATs, EC 2.3.3.15), 2-hydroxyphytanoyl-CoA lyases (2-HPCLs) and 2-succinyl-5-enolpyruvyl-6-hydroxy-3-cyclohexadiene-1-carboxylate synthase (SEPHCHC, MenD). Despite low sequence similarities between sequences of the decarboxylase superfamily of the TEED (~ 20%), their structures are highly similar. The structures consist of three domains, the N- and C-terminal domains are involved in binding of the cofactor ThDP and are named pyrimidine (PYR) and pyrophosphate (PP) binding domain [2,3], respectively. They are separated by a third domain, which is less conserved and adopts different functions in the various enzyme families, e.g. by binding additional cofactors such as ADP [4] and FAD [5] or activators and inhibitors [6]. Due to structural relations between this middle domain and the transhydrogenase domain dIII, this domain is called the TH3 domain [2,3].

Although all ThDP-dependent decarboxylases share the same fold and a similar mechanism utilising the cofactor ThDP, they catalyse a broad range of different reactions involving cleavage and formation of C-C bonds [7-9]. While the decarboxylation of 2-ketoacids [10] and the carboligation of two aldehydes to 2-hydroxy ketones are catalysed by most members of the ThDP-dependent decarboxylases [9], their substrate ranges are different. The well characterised PDC from *Saccharomyces cerevisiae*, BFD from *Pseudomonas putida* and BAL from *Pseudomonas fluorescence* accept a broad variety of substrates [7,11,12], while SEPHCHC-synthase (MenD) is limited to a small number of substrates [13,14]. Additional complexity of C-C bond formation results from the fact that a substrate might be either a donor, which is activated by addition to ThDP in the active site, or an acceptor, which reacts with the ThDP-bound donor, resulting in different products [7,11,12]. Reactions catalysed by members of the structural group of ThDP-dependent decarboxylases include decarboxylation of 2-keto acids, synthesis of various chiral 2-hydroxy ketones by asymmetric benzoin- [11,15] and cross-benzoin condensation [16,17], the racemic resolution of 2-hydroxy ketones via C-C bond cleavage [18], and Stetter-like reactions, e.g. the addition of decarboxylated 2-ketoglutyrate to isochorismate by MenD [19]. With the exception of a few functionally relevant residues that have been identified by comparing sequences and structures of homologous proteins or by mutation experiments, the molecular basis of this biochemical diversity is still unknown. Variants have been developed by rational design and by directed evolution, in order to improve the activity of members of this enzyme family [16,20,21] or to alter substrate specificity [22-28] or stereoselectivity [29-31]. Some functionally relevant amino acids are located in the active site, mediating substrate binding [3], are involved in the activation of ThDP [28] or steer stereoselectivity [29-31], e.g. the *S*-pocket as part of the acceptor binding site, which has been shown to contribute to the stereoselectivity of several members of the decarboxylase superfamily [29-31]. However, due to this complexity, combining results yielded from different variants of different protein families, consolidating results on the function of specific residues and comparing results from different research groups is unfortunately not a straightforward process. An additional challenge in this respect is the identification of homologous positions in sequences of different proteins, in order to allow for their comparison. Amino acid exchanges in enzyme variants are usually identified by a number, signifying the absolute position of the amino acid in the respective protein in combination with the original and the newly introduced amino acid. This method only yields comparable results if the numbering is based on exactly the same sequence. In reality however, published results often are based on slightly different protein sequences, often missing residues at the N-terminus or based on sequences derived from crystal structures. This makes the comparison of results concerning individual residues of one specific protein from different research groups or the comparison of results on homologous proteins manually intensive and prevents the use of automated tools for a large number of sequences. Therefore, an unambiguous numbering scheme for all members of the decarboxylase superfamily would be desirable. The usefulness of a generally accepted numbering scheme was demonstrated for the class A and B enzyme families of β-lactamases [32,33]. Based on structure-guided multisequence alignments of reference sequences [34], a number was assigned to each column of the alignment. Thus, each amino acid could be addressed unambiguously and consistently for all sequences. This numbering scheme is widely applied for the identification of key residues and for the naming of variants [34]. The numbers assigned by this scheme might differ by more than 20 from the absolute amino acid numbering of a respective protein. Without a standard numbering scheme, the systematic comparison of mutations would have to be done manually and would be error-prone. For the same reasons, a standard numbering scheme was established for complementary determining regions (CDRs) of antibodies, thus allowing for a systematic analysis and an unambiguous communication between research groups [35,36]. The numbering schemes were initially based on limited sets of protein sequences and were subsequently refined as more sequence and structure data became available. In order to provide a standard numbering which is independent from the increasing sequence space, a numbering scheme based on one defined reference sequence would be desirable. Due to the low sequence similarity between ThDP-dependent decarboxylases from different homologous families, it would not be reliable to transfer the absolute position numbers of the reference sequence to the residues of any decarboxylase sequence based on pairwise alignments. To handle this challenge, we chose a structure-based and profile-guided approach for the transfer of position numbers. In this work, we present the establishment of a numbering scheme for the ThDP-dependent decarboxylases based on the sequence of the well-documented pyruvate decarboxylase from *S*. *cerevisiae* (PDB: 2VK8 [6], Swissprot: P06169). The numbering scheme was validated by comparing its ability to produce multisequence alignments to the T-Coffee alignment algorithm and by revision of the structural equivalence of positions with the same standard numbers. Using this numbering scheme, the decarboxylase superfamily was systematically analysed for conserved amino acids.

## Results

### Implementation and validation of a standard numbering scheme

A standard numbering scheme for the decarboxylase superfamily of ThDP-dependent enzymes was established using the ThDP-dependent Enzyme Engineering Database (TEED). A profile hidden Markov model was created from a structure-guided multisequence alignment of 16 representative proteins of the decarboxylase superfamily (Table 1). One of the representative proteins, the pyruvate decarboxylase from *S*. *cerevisiae* (*Sc*PDC, Swissprot: P06169, PDB: 2VK8 [6]), was used as the reference sequence for numbering all proteins of the decarboxylase superfamily. In addition, 22 functionally and structurally relevant residues in the sequence of *Sc*PDC were annotated as described in literature [2,5,28,30,31,37-39] (Additional file 1: Table S1). These positions include the highly conserved active site residues E51 (standard numbering) [40-43], the conserved HH motif in PDCs (H114/H115) [28], the GDGX motif 443–446 and the Mg^2+^ binding site N471 [39], as well as more variable regions such as the *S*-pocket residues P26, G27, I476, and Q477 [29-31] and the start and end position of the three decarboxylase domains, the PYR, PP, and the TH3 domain [2]. 

**Table 1:** The set of 16 representative proteins used for establishing a standard numbering scheme

**Protein**	**Organism**	**PDB**-**identifier**
pyruvate decarboxylase	*S. cerevisiae*	2VK8
2-succinyl-5-enolpyruvyl-6-hydroxy-3-cycloheaxdiene-1-carboxylate synthase	*E. coli*	2JLC
pyruvate decarboxylase	*Z. mobilis*	1ZPD
branched-chain keto acid decarboxylase	*L. lactis*	2VBF
benzoylformate decarboxylase	*P. putida*	1BFD
carboxyethylarginine synthase	*S. clavuligerus*	2IHT
cyclohexene-1,2-dione hydrolase	*Azoarcus sp.*	2PGN
oxalyl-CoA decarboxylase	*O. formigenes*	2C31
pyruvate oxidase	*A. viridans*	1V5F
pyruvate dehydrogenase	*E. coli*	3EYA
indolepyruvate decarboxylase	*E. cloacae*	1OVM
acetohydroxyacid synthase	*S. cerevisiae*	1JSC
acetohydroxyacid synthase	*A. thaliana*	1YBH
acetohydroxyacid synthase	*K. pneumoniae*	1OZF
benzaldehyde lyase	*P. fluorescens*	2AG0
glyoxylate carboligase	*E. coli*	2PAN

Of each PDB entry, chain A was used for alignment. It was verified that for all proteins chain A corresponds to the catalytic subunit.

In contrast to the PYR and the PP domain, the secondary structure elements of the TH3 domains of different decarboxylases vary considerably near their N- and C-terminus, thus leading to numerous gaps in the alignment at these positions. Therefore, the start of the TH3 domain was shifted four positions downstream and the end was shifted five positions upstream into regions, which were free of gaps, though sequence conservation was still low.

The absolute amino acid numbers and annotation information were transferred from the reference sequence to the respective positions of all members of the decarboxylase superfamily by aligning them to the profile HMM.

**Figure 1:**
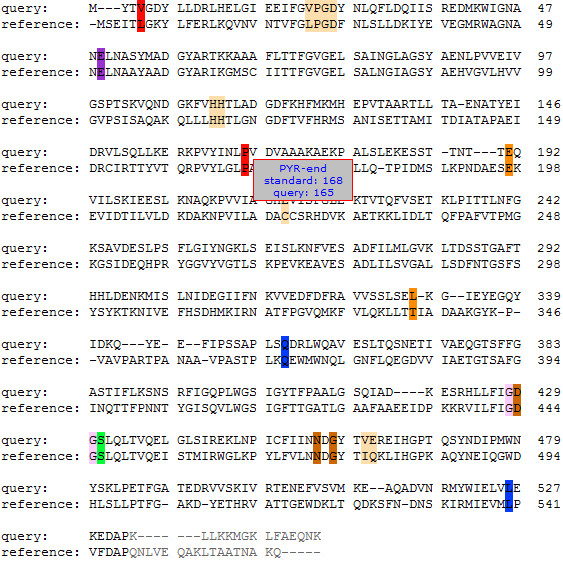
**Alignment of a query sequence and the reference sequence from the web interface of the numbering method.** Alignment of a query sequence (here: branched-chain alpha-ketoacid decarboxylase from *L*. *lactis*, genbank: 75369656) to the reference sequence (*Sc*PDC, swissprot: P06169, PDB: 2VK8). By positioning the cursor on an amino acid (here: proline), the standard numbering (here: 168) as derived from the reference sequence and the absolute numbering of the respective query sequence (here: 165) as well as annotation information (here: end of the PYR domain) are displayed. All residues are highlighted for which annotation information is available in the TEED [1].

A web application was integrated into the web interface of the TEED ( http://www.TEED.uni-stuttgart.de) to provide public access to the numbering tool. Upon submission of a single query sequence or a list of sequences in FASTA format, the standard numbering is applied and the sequence including the numbering and annotations for each amino acid can be downloaded (Figure 1; a description of the file format is given in the Additional file 1, a sample is given in the Additional file 2).

**Figure 2:**
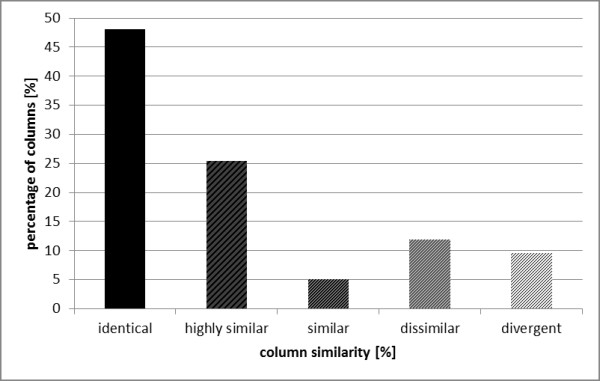
**Analysis of accordance of two multisequence alignments.** The comparison of columns of two multisequence alignments of 15 sequences using the numbering method and T-Coffee revealed five types of column similarity. 48% of the investigated columns were identical in both alignments, 25% of the columns were “highly similar” (up to 3 mismatches out of 15 sequences), 5% were “similar” (4 – 8 mismatches), 12% of the columns had 9 to 12 mismatches and are therefore called “dissimilar” and 10% of the columns showed more than 12 mismatches (“divergent”).

The accuracy of the HMM-based alignment was compared to a multisequence alignment using T-Coffee [44] by aligning the reference sequence *Sc*PDC and 15 sequences from the decarboxylase family for which structural information was available but which were not part of the set of representative proteins. To determine the differences between the HMM-based alignment and the T-Coffee alignment, all columns were compared between the two alignments and a similarity score was assigned to each column (Additional file 3). Alignment columns were "identical" if both alignment algorithms placed the same residues for all sequences into the respective columns; "highly similar" if the two alignments differed in 1–3 sequences; "similar" if 4–8 mismatches were observed; "dissimilar" if 9 – 12 sequences differed at the respective position; "divergent" if the alignments differed in 13 – 15 of the 15 sequences. As a result, 73% of all columns were identical or highly similar in both alignments (Figure 2). For those columns which deviated considerably between the two alignments (dissimilar or divergent columns), a structural comparison revealed that in almost all cases the HMM-based alignment represented the structural equivalence better than the multisequence alignment by T-Coffee (Additional file 1: Figures S1, and S2). In addition, it was verified that all 22 functionally relevant positions were aligned correctly (Additional file 1: Table S1). 

### Identification of conserved residues and domain boundaries

After having applied a standard numbering scheme for all 3000 members of the decarboxylase superfamily, the respective protein sequences were systematically analysed for the occurrence of amino acids at corresponding positions. Four groups of positions with different characteristics of conservation were found.

The first group includes 6 positions which were conserved in more than 90% of all members of the decarboxylase superfamily, while no other amino acid occurred in more than 1% of the sequences: Position 27 (standard numbering) in the *S*-pocket which was glycine in 91% of all members of the decarboxylase superfamily, position 443 in the GDGX motif which was glycine in 98% of all decarboxylases, and four highly conserved positions which have not yet been identified as being of functional or structural relevance: positions 58 (alanine in 96% of the sequences), 94 (proline in 91% of the sequences), 219 (glycine in 91% of the sequences), and 286 (glycine in 97% of the sequences). Thus, 6 positions (mostly glycine residues) are highly conserved in almost all members of the decarboxylase superfamily.

The second group includes 3 positions in which one amino acid was found in a majority of more than 90% of all members of the decarboxylase superfamily and a different amino acid in a minority (> 3%) of all sequences. The most conserved position was the active site residue Glu 51. This conserved glutamic acid was found in 94% of all sequences, while 3% have a valine in this position. D444 of the GDGX motif was conserved in 91% of all cases, while 7% have a glutamic acid in this position. At position 280, aspartic and glutamic acid were found in 90% and 4%, respectively, of all members of the decarboxylase superfamily. Thus, this group includes positions which seem to be characteristic for a distinct subgroup of this superfamily.

The third group encompasses variable positions which are known to be involved in substrate recognition or catalysis. In positions 114 and 115, the majority of all members of the decarboxylase superfamily have a phenylalanine (58%) and a glutamine (81%), respectively, while a minority, predominantly PDCs, show histidine (15% and 12%, respectively) in these positions. These histidines have been referred to as the HH-motif in the PDC family [28]. A functionally relevant, though highly variable site, is the *S*-pocket which contributes to the stereo selectivity of decarboxylases [29-31]. Two positions, 476 and 477, which were shown to contribute to the *S*-pocket or the entrance of the *S*-pocket, were highly variable in all members of the decarboxylase superfamily. In standard position 476 most members of the decarboxylase superfamily show a methionine (42%) or an isoleucine residue (18%), respectively, while standard position 477 is occupied by valine (45%) or isoleucine (20%), respectively.

The fourth group included the domain boundaries of the three protein domains PYR, PP and the TH3 domain. Identification of the domain boundaries can be easily accomplished when structural information is available, whereas an identification of domain boundaries based on the amino acid sequence alone is not straightforward due to the low sequence similarity in the loop regions connecting the three domains. However, alignments using the profile HMM revealed several conserved positions: the start of the PYR domain (standard numbering 6) is indicated by a conserved glycine (in 44% of all sequences), while its end (position 168) is highly conserved (proline in 87% of all cases). Similarly, the PP domain starts at position 367 (proline in 54% of all sequences) and ends at position 540 (valine in 37% of all sequences). These four positions coincided well with the start and end of the ThDP-binding fold. In contrast, the start and end positions of the TH3 domain were highly variable. Therefore, two positions further inside the TH3 domain were selected to characterise the start and the end of this domain: positions 197 (aspartic acid in 18% of all cases) and 336 (lysine in 17% of all sequences). Despite the low sequence similarity in the boundary region, the assignment of standard numbers was consistent with the results from a structural superimposition.

Furthermore, the regions around the 9 highly conserved positions of group 1 and 2 were investigated concerning sequence conservation in order to investigate the presence of sequence motifs. With the exception of position 27 (standard numbering), their surrounding regions were sufficiently conserved to allow for the derivation of sequence motifs. The region around residue G443 is already known as the **G**DGX_24,27_N-motif [45]. In order to analyse the specificity and the precision of the remaining motifs for the decarboxylase superfamily, they were used in a motif search against the non-redundant NCBI database, while an updated version of the TEED (not yet published) served as positive control [1]. The motif [DHN]_50_-E_51_-[AEGLQ]_52_-[AGNSTV]_53_-[AGLMV]_54_-[AGISTV]_55_-[FHLMY]_56_-[AFILM]_57_-A_58_, which was derived from the region around the conserved positions 51 and 58, showed similar sensitivity (0.65) and precision (0.27) as the PROSITE pattern PS00187, which is an extended version of the GDGX_24,27_N-motif and was described as a conserved motif of POXs (EC 1.2.3.3), PDCs (EC 4.1.1.1), AHASs (EC 2.2.1.6), BFDs and indolepyruvate decarboxylases (IPDCs, EC 4.1.1.74) [46-48] (sensitivity: 0.59, precision: 0.42). This motif is part of an α-helix, which is involved in the formation of the active site. In addition, the motif surrounding position 280 had at least similar precision and sensitivity for ThDP-dependent decarboxylases as the simple GDGX_24,27_N–motif [45] (data not shown). Thus, a second motif [DE]_280_-[ACFLTV]_281_-[ILMV]_282_-[FILV]_283_-[ACGLMNSTV]_284_-[AFILV]_285_-G_286_ was identified with 5 predominantly hydrophobic amino acids between two highly conserved positions D/E280 and G286, which form the vertices of the loops connecting a central β-strand of the TH3 domain to the adjacent α-helices. The remaining motifs were less specific and sensitive for the identification of ThDP-dependent decarboxylases.

### Application of the numbering scheme to experimentally characterized positions

An extensive literature search yielded 22 positions which were experimentally well characterized in five different proteins (*Sc*PDC, *Ap*PDC, *Zm*PDC, *Pf*BAL and *Pp*BFD) and shown to be of relevance to substrate specificity and/or activity. The numbering scheme was exemplarily applied to the respective sequences in order to compare the annotation information from the literature. Several equivalent positions in different proteins were shown to have different absolute numbers (Additional file 1: Table S2). An influence on the decarboxylase activity was shown for the residues D28 of *Sc*PDC, D27 of *Zm*PDC and A28 of *Pf*BAL, each corresponding to standard position 28. Furthermore, structural and functional equivalence was shown for A28 in *Pf*BAL and S26 in *Pp*BFD. Similarly, positions 114 and 115, which were described as the HH-motif of pyruvate decarboxylases (Additional file 1: Table S2) [28] are structurally and functionally identical in different PDCs, but differ in their absolute position numbers. The mutations W388A,I in *Ap*PDC were shown to reduce stereoselectivity while the mutations W392A,I,M of *Zm*PDC led to an improved carboligation activity. However, both positions are structurally equivalent and are addressed with standard number 392. Functional relevance is also described for position 477 (standard number) in *Sc*PDC, *Ap*PDC and *Zm*PDC. All mutations of the respective residues (E477Q in *Sc*PDC, E469G in *Ap*PDC and E473D,Q) revealed an impact on the decarboxylation reaction [23-25,29]. The examination of these five examples and the differences between the absolute and the standard numbers of functionally equivalent positions showed, that the presented numbering scheme for the ThDP-dependent decarboxylases eases the communication on variants and the comparison of functionally relevant positions. The assignment of standard numbers to positions of different homologous proteins furthermore simplifies the prediction of the impact of mutations at equivalent positions.

## Discussion

A standard numbering scheme has been established for the structural superfamily of ThDP-dependent decarboxylases, as it has been done previously for two protein families, the β-lactamase family and the complementary determining regions of antibodies [34,35]. A standard numbering scheme for a protein family enables an unambiguous communication between research groups about corresponding positions in different proteins and supports the automated systematic analysis of sequences and the classification of proteins into sub-groups [49]. In principle, a numbering scheme could be established by performing pairwise alignments of each sequence of the protein family to a reference sequence. However, although structurally conserved, the superfamily of ThDP-dependent decarboxylases shows only low sequence similarity. As a consequence, pairwise alignments are in general not reliable. As an alternative, multisequence alignment methods were successfully applied to align homologous proteins with low sequence similarity [50]. By performing a multisequence alignment of all sequences of the decarboxylase superfamily, the numbering of a reference sequence could be transferred to each aligned decarboxylase sequence. However, a new alignment has to be calculated for each new sequence to be included. Calculating multisequence alignments of many thousands of sequences with low sequence similarity are not only computationally intensive, but more importantly, they lack robustness, because the alignment might change upon inclusion of additional sequences. In contrast, profile hidden Markov models (HMM) based on a structure-driven alignment are a robust description of protein families and allow the user to align new sequences to an existing multisequence alignment [51]. By alignment of a sequence to a profile built from a set of representative proteins, the numbering can be transferred from the reference sequence to a query sequence. However, the quality of the numbering depends on the quality of the profile. Therefore, the proteins in the profile HMM were carefully selected. From each of the sixteen families with structural information, a representative protein was selected for a structure-guided alignment [52] to guarantee the structural equivalence in the reference profile. Because some members of the decarboxylase superfamily show activation upon binding of a substrate at a second (allosteric) binding site (e.g. *Sc*PDC) [6] which leads to conformational changes, the set of reference proteins only contained decarboxylases which show no substrate activation or which have been crystallized in complex with an allosteric activator. Thus, only structures of active enzymes were compared. The alignment was further manually refined in order to improve consistency and robustness. Since the presented numbering scheme is aimed to compare structurally equivalent positions, the method depends on structural similarity of the proteins in the corresponding family. Accordingly, the method can be adapted to other protein families matching this requirement globally or at least in structurally conserved domains.

By establishing a standard numbering scheme for the ThDP-dependent decarboxylase superfamily, the unambiguous identification, numbering, and analysis of functionally and structurally relevant residues was possible. The analysis of conserved positions in the protein family of ThDP-dependent decarboxylases revealed that the previously observed substitution of the active site glutamate by valine in members of the glyoxylate carboligase family at standard position 51 [43,53] is indeed characteristic of the entire family, which indicates a different mechanism in glyoxylate carboligases [53]. It could also be shown that the active site “HH-motif” which has been described for various members of the decarboxylase superfamily [28] is highly specific for only a small number of decarboxylases, the pyruvate decarboxylases, indolepyruvate decarboxylases, and phenylpyruvate decarboxylases, and is not present in the majority of the enzymes. The four highly conserved glycine residues at standard positions 27, 219, 286 and 443 are all located between the C-cap of a β-strand and the N-cap of an α-helix of β-α-β supersecondary structure elements, which has been shown to be a typical pattern for α-β units [54]. These elements presumably are relevant for the correct folding of the ThDP-dependent decarboxylases.

The assignment of standard numbers to experimentally well characterized positions allows for an easy comparison of positions between different proteins and different organisms regarding their structurally equivalence. This was demonstrated by an in-depth analysis of five different members of the decarboxylase superfamily (Additional file 1: Table S2). Several positions were identified which share the same standard numbers, show similar functional influence and are structurally equivalent, but deviate in their absolute position numbers by up to 8 positions. Prediction of the functional influence of mutations in homologous sequences based on the absolute position numbers of given sequences is not straightforward, but becomes feasible using a standard numbering scheme. Thus, new sequence motifs were found by systematically analysing the amino acid distribution at each position of all members of the ThDP-dependent decarboxylase family. A new family-specific sequence motif was derived from the conserved region near the catalytic glutamic acid at position 51 (standard position) and the conserved alanine at position 58 (standard position). The respective motif was shown to be as sensitive and precise for the ThDP-dependent decarboxylases as the PROSITE pattern PS00187, but due to the defined E51, it cannot be used to identify glyoxylate carboligases, which have a valine at the respective position [53]. In addition, despite the higher variability of the TH3 domain in comparison to the PYR and the PP domain, the sequence of a β-strand found in the TH3 domain (standard positions 280–286) consists of a conserved motif. In contrast to the previously mentioned motif, this region is not part of the active site but is presumably relevant for the structure or regulation of the protein. The adjacent loop region 286–304 was described as a part of the activation cascade of pyruvate decarboxylases, since this loop shows structural rearrangement upon binding of an activator at the effector binding site at standard position 221 [6].

## Conclusions

By introducing a robust and reliable numbering scheme for the family of ThDP-dependent decarboxylases, we provided a frame of reference for this diverse protein family. Besides being a reliable tool to identify and number residues and domain boundaries for the superfamily of ThDP-dependent decarboxylases, the presented implementation of a numbering scheme is generic and can be adapted to other protein families as well. The usefulness and reliability of the presented numbering method was demonstrated for various examples.

## Methods

### Reference alignment and position number assignment

16 representative members of the decarboxylase superfamily were selected from the ThDP-dependent Enzyme Engineering Database [1] by three criteria: 1) Only proteins with known crystal structure were chosen for the reference alignment. From each of the 16 homologous families that contain structure information, one member was selected. 2) Some decarboxylases show activation upon binding of a substrate molecule to an allosteric binding site which leads to conformational changes. In these cases only structures were chosen which were crystallized in complex with a bound substrate or a substrate analogue. 3) For homologues families with more than one structure entry matching these criteria, the structure with the highest resolution was selected.

From these 16 representative proteins, a structure-guided multisequence alignment was created by STAMP [52]. This reference alignment was manually refined to align secondary structure elements and thus to reduce the number of gaps scattered in the alignment (Additional file 4). A family specific profile hidden Markov model was derived from the reference alignment by HMMER [55].

The sequence of the pyruvate decarboxylase from *S*. *cerevisiae* (PDB: 2VK8 [6], Swissprot: P06169, EC: 4.1.1.1) was chosen as the reference sequence, because it is a widely applied and well characterized ThDP-dependent enzyme [6-8,56]. Standard position numbers were assigned by aligning the sequence of each member of the decarboxylase superfamily against the profile HMM and by subsequently transferring the absolute position numbers of the reference sequence to the corresponding positions of the respective decarboxylase sequence.

### Web tool

An open access web application is provided to allow users to assign standard position number for decarboxylase sequences ( http://www.teed.uni-stuttgart.de). After submitting a query sequence, a BLAST search against a database of members of the structural group of decarboxylases from the TEED [1] is performed. Only query sequences with an E-value less than 10^-10^ are accepted to guarantee for a reliable sequence alignment. Then the query sequence is aligned to the reference alignment using the profile HMM, and the absolute position numbers of the reference sequence are transferred to the query sequence. Finally, annotation information of the TEED such as catalytic residues, domain boundaries, or activator binding sites is transferred to the respective positions of the query sequence.

## Authors’ contributions

CV participated in the development of the algorithm of the numbering scheme, carried out the programming and the design of the reference alignment and contributed to the analysis of the amino acid frequencies. MW participated in the development of the algorithm, carried out the validation, drafted the manuscript and performed the alignment comparisons. MP selected the reference sequences and helped in the application of the numbering scheme on experimentally characterized positions. JP supervised the study. All authors read and approved the final manuscript.

## Supplementary Material

Additional file 1 Figures S1 and S2, Tables S1 and S2, Description of the nvw file format. Click here for file

Additional file 2 **nvw file of the pyruvate decarboxylase from *S. cerevisiae*.** Click here for file

Additional file 3 Comparison of alignments generated using the numbering method and T-Coffee. Click here for file

Additional file 4 Reference alignment for the standard numbering method. Click here for file
