# Supplementary material for: A standard numbering scheme for thiamine diphosphate-dependent decarboxylases
Source: BMC Biochem. 2012 Nov 17;13:24. doi: 10.1186/1471-2091-13-24 (PMC3534367; doi:10.1186/1471-2091-13-24)
Supplement: Additional file 1 — Figures S1 and S2, Tables S1 and S2, Description of the nvw file format. [file 1471-2091-13-24-S1.pdf]

Supplementary material

A standard numbering scheme for thiamine diphosphate-dependent decarboxylases

Constantin Vogel[1,†]

Email: constantin.vogel@itb.uni-stuttgart.de

Michael Widmann[1,†]

Email: michael.widmann@itb.uni-stuttgart.de

Martina Pohl[2]

Email: ma.pohl@fz-juelich.de

Jürgen Pleiss[1*]

[*] Corresponding author

Email: juergen.pleiss@itb.uni-stuttgart.de

[1] Institute of Technical Biochemistry, University of Stuttgart, Allmandring 31, Stuttgart 70569, Germany

[2] IBG-1: Biotechnology, Forschungszentrum Jülich GmbH, Jülich 52425, Germany

[†] Equal contributors.

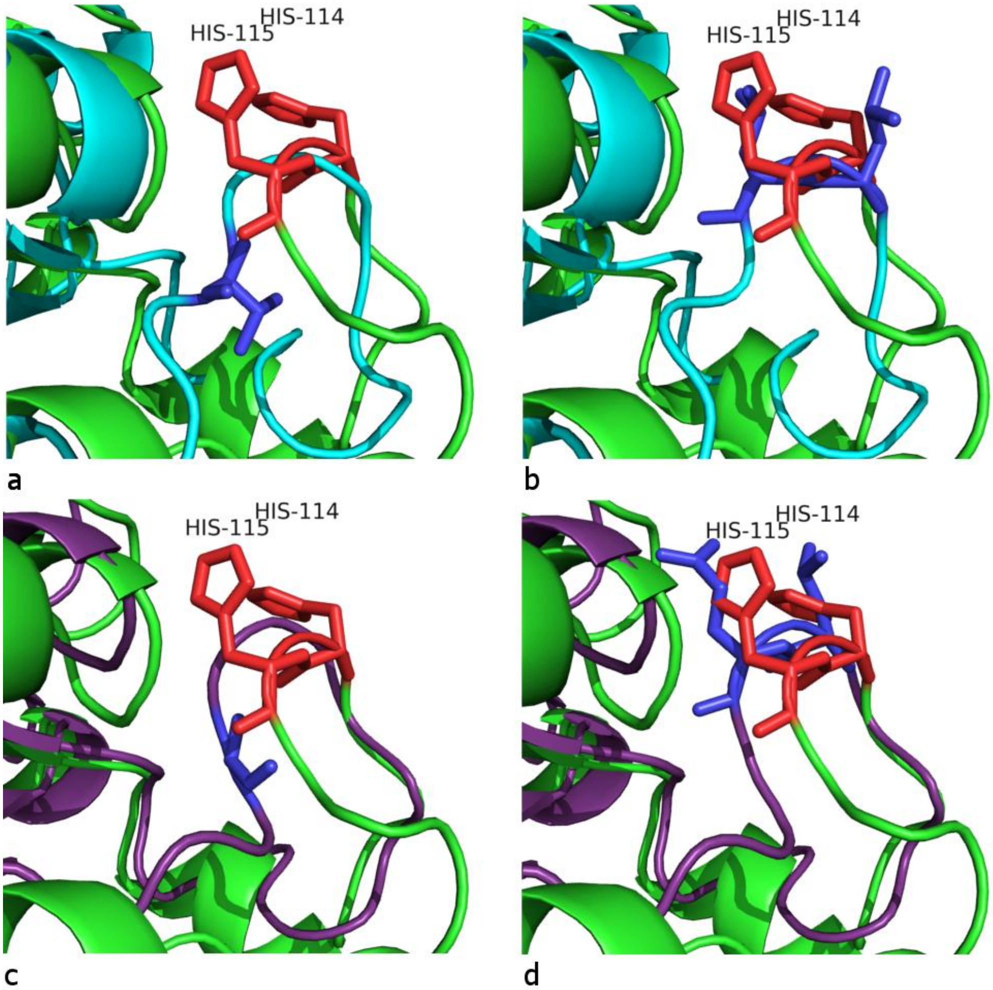

Figure S1a-d – “Dissimilar” positions 114 and 115 (HH-motif) of alignment comparison between T-Coffee and the alignment method using the standard numbering scheme

“Dissimilar” positions 114 and 115 (HH-motif) of alignment comparison between T-Coffee and the alignment method using the standard numbering scheme. The numbering scheme based alignment led to an accurate prediction of the residues belonging to the HH-motif of the pyruvate decarboxylases (Fig. S1b and S1d), while the positions, which were aligned against the positions 114-115 of the pyruvate decarboxylase by T-Coffee, do not show structural correspondence (Fig, S1a and S1c). The superimposition shows overlays of the reference structure (pyruvate decarboxylase from *S. cerevisiae*; PDB: 2VK8; green) with the benzoylformate decarboxylase from *P. putida* (PDB: 1MCZ; cyan, S1a and S1b) and with the benzaldehyde lyase from *P. fluorescens* (PDB: 3D7K; purple, S1c and S1d).

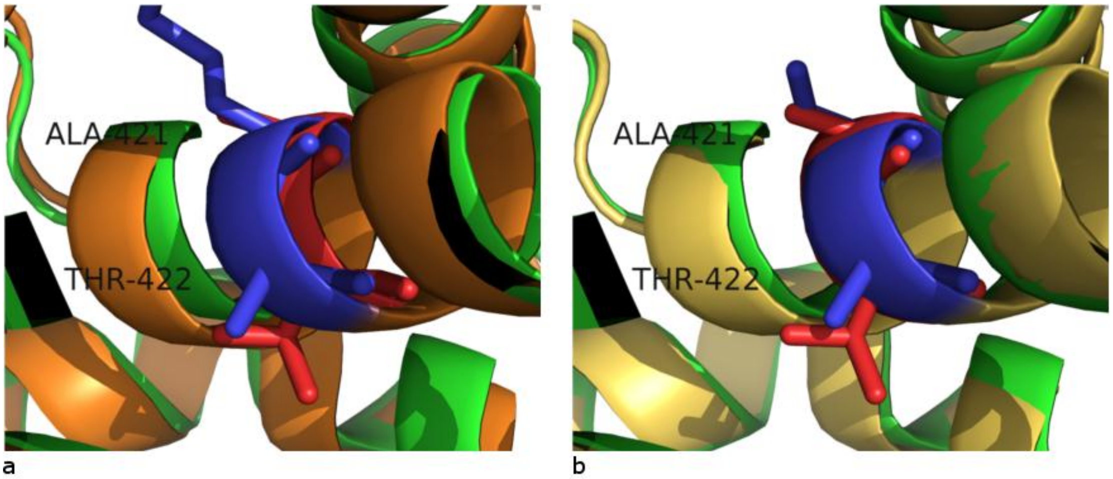

Figure S2a,b – Divergent positions 421 and 422 in comparison of the alignment methods

Positions 421 and 422 are part of the PP domain. The alignment method using the numbering scheme was able to provide a perfect match in the structural superimposition of the reference structure (pyruvate decarboxylase from *S. cerevisiae*; PDB: 2VK8; green) and the structures of cyclohexane-1,2-dione hydrolase from *Azoarcus sp.* (PDB: 2PGO; orange, Fig. S2a) and the pyruvate decarboxylase from *A. pasteurianus* (PDB: 2VBI; yellow, Fig. S2b).

Table S1 – 22 positions of interest in ThDP-dependent decarboxylases including positions with known functional relevance and the assigned domain boundaries. Concerning the function of specific residues see also Tab. S2.

reference position		6	25	26	27	28	51	114	115	168	197	221
function		PYR start	S pocket	S pocket	S pocket	S pocket	cofactor activation	active site	active site	PYR end	TH3 start	activator binding
protein	acc nr											
ApPDC	tr|Q8L388	V5	V24	G25	G26	D27	E50	H113	H114	A167	L195	K219
PpBFDC	gi|3915757	V4	N23	P24	G25	S26	E47	L109	L110	P159	N186	D210
EcAHAS I	gi|16131541	G15	I34	P35	G36	G37	E60	F122	Q123	P171	S197	G221
EcAHAS II	gi|33112641	G3	Y22	P23	G24	G25	E47	F109	Q110	P158	P182	G206
PpBAL	gi|1705519	G6	L25	H26	G27	A28	E50	L112	Q113	P162	D190	E214
EcGXC	gi|84028422	A6	V25	P26	G27	A28	V51	F114	Q115	P163	S189	G213
LlkdcA	gi|75369656	V4	V23	P24	G25	D26	E49	H112	H113	P165	E191	E215
EcMenD	sp|P17109	W10	A29	P30	G31	S32	E55	N117	Q118	P169	E207	
most frequent aa		G 44 %	Y 33 %	P 74 %	G 91 %	G 39 %	E 94 %	F 58 %	Q 81 %	P 87 %	D 18 %	G 71 %
		V 13 %	I 19 %	V 6 %		D 18 %	V 3 %	H 15 %	H 12 %	A 3 %	E 9 %	L 4 %

reference position		336	367	443	444	445	446	471	473	476	477	540
function		TH3 end	PP start	**G**DGX	G**D**GX	GD**G**X	GDG**X**	Mg+ binding	Mg+ binding	S pocket	S pocket	PP end
protein	acc nr											
ApPDC	tr|Q8L388	K334	N363	G434	D435	G436	S437	N462	G464	I467	E468	I530
PpBFDC	gi|3915757	L328	P355	G427	D428	G429	S430	N455	T457	A460	L461	T523
EcAHAS I	gi|16131541	L337	H370	G443	D444	G445	S446	N471	A473	L476	V477	I540
EcAHAS II	gi|33112641	P324	A354	G427	D428	G429	S430	N455	R457	M460	V461	I524
PpBAL	gi|1705519	A332	P372	G447	D448	G449	S450	N475	S477	A480	T481	V544
EcGXC	gi|84028422	V332	P372	G445	D446	F447	D448	N473	Y475	L478	I479	L555
LlkdcA	gi|75369656	L330	Q356	G428	D429	G430	S431	N456	G458	V461	E462	L526
EcMenD	sp|P17109	P338	E369	G441	D442	L443	S444	N469	G471	I474	F475	V537
most frequent aa		L 17 %	P 54 %	G 98%	D 91 %	G 70 %	S 52 %	N 89 %	G 34 %	M 42 %	V 45 %	V 37 %
		A 13 %	E 8 %		E 7 %	A 9 %	G 20 %	D 9 %	Y 12 %	I 18 %	I 20 %	I 22 %

Table S2 – Functionally relevant positions in selected ThDP-dependent decarboxylases found in literature

Protein	Organism	PDB	Mutation	Standard position	Effect	Reference
PDC	*S. cerevisiae*	1QPB	D28A,N	28	Is involved in rate limiting steps of decarboxylation; D28N,A catalyzes formation of S-acetolactate as the major product, besides acetoin.	[1-3]
			E51D,Q,A	51	Stabilizes the cofoactor ThDP; E51Q decreases the catalytic activity, E51A leads to inactivity.	[4, 5]
			E91D,Q,A	91	Stabilizes the zwitter-ionic enamine intermediate. 10^9 fold acceleration of decarboxylation of hydroxyl-benzyl-ThDP; part of the substrate activation cascade; charge necessary for activity.	[6, 7]
			H92	92	Part of the substrate activation cascade.	[7]
			H114F	114	Is involved in rate limiting steps of decarboxylation.	[1-3]
			H115F	115	Is involved in rate limiting steps of decarboxylation.	[1-3]
			C221S,A	221	Important residue for allosteric activation by pyruvate or pyruvate amide.	[8-12]
			W412F,A	412	Impact on substrate activation, Ala-variant: very much reduced substrate activation.	[7, 13]
			E477Q	477	Is involved in rate limiting steps of decarboxylation.	[1-3]
	A. pasteurianus	2VBI	W388A,I	392	Alanin-variant shows reduced *R* resp. *S*-selectivity, although S-pocket is closed by E469. Suggests alternative S pathway.	[14]
			E469G	477	Mutation opens *S*-pocket and (*S*)-2-hydroxyketones are formed; almost no decarboxylase activity.	[14]
	Z. mobilis	1ZPD	D27E,N,A	28	Strongly reduced decarboxylase activity; D27A shows weak acetolactate forming activity.	[15-17]
			E50Q	51	Polarizes pyrimidine ring.	[16]
			H113Q,K,R	114	H113Q inactive for decarboxylation.	[16, 18]
			H114Q,A	115	H114Q k_{cat} slower.	[16, 18]
			W392A,I,M	392	Improved carboligase activity.	[19-22]
			I472A	476	Altered substrate range.	[23]
			E473D,Q	477	Decarboxylation of α-lactyl-ThDP and protonation of HE-ThDP is reduced, but carboligase activity is increased; cofactor binding in E473Q is tighter than in wt*Zm*PDC	[15, 16, 24-26]
			I476F	480	Altered substrate range.	[23]
BAL	*P. fluorescenz*	2AG0	A28S	28	Introduces weak decarboxylase activity into BAL; reduces ligase activity; A28 corresponds to S26 in BFD.	[27-30]
BFD	*P. putida*	1BFD	S26	28	Reduces decarboxylase activity. Increased K_M for benzoylformate. S26 corresponds to A28 in BAL.	[30]
			H281A	292	Improved benzoin forming activity.	[31, 32]
			A460I	476	Altered substrate range.	[23]
			L461A,G	477	Residue determines size of *S*-pocket; Mutation decreases decarboxylase activity	[33]

Description of the "nvw" file format for biological sequences using a reference sequence based standard numbering scheme

In order to provide a file format which is able to display amino acid sequences and position specific standard numbers, we developed the "nvw" file format. It contains a header, a title, optional annotation information and the numbered sequence. The header is marked by the '#'-sign and can span several lines. Each "nvw" file can contain one title, which has to be placed between two '//'-signs. The optional lines starting with '#ANNODESC' and '#ANNOPOS' provide additional information about the position and the description of annotation in the numbered sequence. The '#ANNODESC' lines defined the description of annotations and must consist of the following pattern:

#ANNODESC [description id] [description] [color]

Each annotation description must have a unique description id and a distinct color in the hexadecimal color code to allow highlighting of the respective positions in multisequence alignments, which can be generated based on "nvw"-files.

The '#ANNOPOS' lines define the position of given annotations on the numbered sequence and consist of:

#ANNOPOS [standard position number] [description id]

The sequence and the standard numbers have to be given in vertical columns of maximum 50 rows. The supplementary file "example_2VK8.nvw" shows the sequence of the *Sc*PDC (PDB: 2VK8) numbered using the presented numbering scheme.

References

1. Liu M, Sergienko EA, Guo F, Wang J, Tittmann K, Hübner G, Furey W, Jordan F: **Catalytic acid-base groups in yeast pyruvate decarboxylase. 1. Site-directed mutagenesis and steady-state kinetic studies on the enzyme with the D28A, H114F, H115F, and E477Q substitutions.** *Biochemistry* 2001, **40:**7355-7368.
2. Sergienko EA, Jordan F: **Catalytic acid-base groups in yeast pyruvate decarboxylase. 3. A steady-state kinetic model consistent with the behavior of both wild-type and variant enzymes at all relevant pH values.** *Biochemistry* 2001, **40:**7382-7403.
3. Sergienko EA, Jordan F: **Catalytic acid-base groups in yeast pyruvate decarboxylase. 2. Insights into the specific roles of D28 and E477 from the rates and stereospecificity of formation of carboligase side products.** *Biochemistry* 2001, **40:**7369-7381.
4. Killenberg-Jabs M, König S, Eberhardt I, Hohmann S, Hübner G: **Role of Glu51 for Cofactor Binding and Catalytic Activity in Pyruvate Decarboxylase from Yeast Studied by Site-Directed Mutagenesis** *Biochemistry* 1997, **36:**1900-1905.
5. Candy JM, Koga J, Nixon PF, Duggleby RG: **The role of residues glutamate-50 and phenylalanine-496 in Zymomonas mobilis pyruvate decarboxylase.** *Biochem J* 1996.
6. Jordan F, Li H, Brown A: **Remarkable stabilization of zwitterionic intermediates may account for a billion-fold rate acceleration by thiamin diphosphate-dependent decarboxylases.** *Biochemistry* 1999, **38:**6369-6373.
7. Li H, Furey W, Jordan F: **Role of glutamate 91 in information transfer during substrate activation of yeast pyruvate decarboxylase.** *Biochemistry* 1999, **38:**9992-10003.
8. Baburina I, Dikdan G, Guo F, Tous GI, Root B, Jordan F: **Reactivity at the substrate activation site of yeast pyruvate decarboxylase: inhibition by distortion of domain interactions.** *Biochemistry* 1998, **37:**1245-1255.
9. Baburina I, Gao Y, Hu Z, Jordan F, Hohmann S, Furey W: **Substrate activation of brewers' yeast pyruvate decarboxylase is abolished by mutation of cysteine 221 to serine.** *Biochemistry* 1994, **33:**5630-5635.
10. Joseph E, Wei W, Tittmann K, Jordan F: **Function of a conserved loop of the beta-domain, not involved in thiamin diphosphate binding, in catalysis and substrate activation in yeast pyruvate decarboxylase.** *Biochemistry* 2006, **45:**13517-13527.
11. Lu G, Dobritzsch D, Baumann S, Schneider G, König S: **The structural basis of substrate activation in yeast pyruvate decarboxylase. A crystallographic and kinetic study.** *Eur J Biochem* 2000, **267:**861-868.
12. Wang J, Golbik R, Seliger B, Spinka M, Tittmann K, Hübner G, Jordan F: **Consequences of a modified putative substrate-activation site on catalysis by yeast pyruvate decarboxylase.** *Biochemistry* 2001, **40:**1755-1763.
13. Li H, Jordan F: **Effects of substitution of tryptophan 412 in the substrate activation pathway of yeast pyruvate decarboxylase.** *Biochemistry* 1999, **38:**10004-10012.
14. Rother D, Kolter G, Gerhards T, Berthold Siöberg CL, Gauchenova E, Knoll M, Pleiss J, Müller M, Schneider G, Pohl M: **(*S*)-Selective mixed benzoin

condensation by structure-based design of the pyruvate decarboxylase from *Acetobacter pasteurianus*. *ChemCatChem* 2011, **3:**1587-1596.

15. Chang AK, Nixon PF, Duggleby RG: **Aspartate-27 and glutamate-473 are involved in catalysis by *Zymomonas mobilis* pyruvate decarboxylase.** *Biochem J* 1999, **339:**255-260.
16. Huang CY, Chang AK, Nixon PF, Duggleby RG: **Site-directed mutagenesis of the ionizable groups in the active site of *Zymomonas mobilis* pyruvate decarboxylase: effect on activity and pH dependence.** *Eur J Biochem* 2001, **268:**3558-3565.
17. Wu YG, Chang AK, Nixon PF, Li W, Duggleby RG: **Mutagenesis at Asp27 of pyruvate decarboxylase from *Zymomonas mobilis*. Effect on its ability to form acetoin and acetolactate.** *Eur J Biochem* 2000, **267:**6493-6500.
18. Schenk G, Leeper FJ, England R, Nixon PF, Duggleby RG: **The role of His113 and His114 in pyruvate decarboxylase from *Zymomonas mobilis*.** *Eur J Biochem* 1997, **248:**63-71.
19. Bruhn H, Pohl M, Grötzinger J, Kula MR: **The replacement of Trp392 by alanine influences the decarboxylase/carboligase activity and stability of pyruvate decarboxylase from *Zymomonas mobilis*.** *Eur J Biochem* 1995, **234:**650-655.
20. Goetz G, Iwan P, Hauer B, Breuer M, Pohl M: **Continuous production of (*R*)-phenylacetylcarbinol in an enzyme-membrane reactor using a potent mutant of pyruvate decarboxylase from *Zymomonas mobilis*.** *Biotechnol Bioeng* 2001, **74:**317-325.
21. Iwan P, Goetz G, Schmitz S, Hauer B, Breuer M, Pohl M: **Studies on the continuous production of (*R*)-(-)-phenylacetylcarbinol in an enzyme-membrane reactor.** *Journal of molecular catalysis B, Enzymatic* 2001, **11:**387-396.
22. Pohl M: **Protein design on pyruvate decarboxylase (PDC) by site-directed mutagenesis. Application to mechanistical investigations, and tailoring PDC for the use in organic synthesis.** *Adv Biochem Eng Biotechnol* 1997, **58:**15-43.
23. Siegert P, McLeish MJ, Baumann M, Iding H, Kneen MM, Kenyon GL, Pohl M: **Exchanging the substrate specificities of pyruvate decarboxylase from *Zymomonas mobilis* and benzoylformate decarboxylase from *Pseudomonas putida*.** *Protein Eng Des Sel* 2005, **18:**345-357.
24. Breslow R: **Rapid deuterium exchange in tetrazolium salts.** *J Am Chem Soc* 1957, **79:**1762.
25. Meyer D, Neumann P, Parthier C, Friedemann R, Nemeria N, Jordan F, Tittmann K: **Double duty for a conserved glutamate in pyruvate decarboxylase: evidence of the participation in stereoelectronically controlled decarboxylation and in protonation of the nascent carbanion/enamine intermediate.** *Biochemistry* 2010, **49:**8197-8212.
26. Meyer D, Pohl M, Müller M, Tittmann K: **Conversion of pyruvate decarboxylase into an enantioselective carboligase with biosynthetic potential.** *J Am Chem Soc* 2011, **133:**3609-3616.
27. Brandt GS, Kneen MM, Petsko GA, Ringe D, McLeish MJ: **Active-site engineering of benzaldehyde lyase shows that a point mutation can confer both new reactivity and susceptibility to mechanism-based inhibition.** *J Am Chem Soc* 2010, **132:**438-439.

28. Brandt GS, Nemeria N, Chakraborty S, McLeish MJ, Yep A, Kenyon GL, Petsko GA, Jordan F, Ringe D: **Probing the active center of benzaldehyde lyase with substitutions and the pseudosubstrate analogue benzoylphosphonic acid methyl ester.** *Biochemistry* 2008, **47:**7734-7743.
29. Janzen E, Müller M, Kolter-Jung D, Kneen MM, McLeish MJ, Pohl M: **Characterization of benzaldehyde lyase from *Pseudomonas fluorescens* - a versatile enzyme for asymmetric C-C-bond formation.** *Bioorg Chem* 2006, **34:**345-361.
30. Kneen MM, Pogozheva ID, Kenyon GL, McLeish MJ: **Exploring the active site of benzaldehyde lyase by modeling and mutagenesis.** *Biochim Biophys Acta* 2005, **1753:**263-271.
31. Dünkelmann P, Kolter-Jung D, Nitsche A, Demir AS, Siegert P, Lingen B, Baumann M, Pohl M, Müller M: **Development of a donor-acceptor concept for enzymatic cross-coupling reactions of aldehydes: the first asymmetric cross-benzoin condensation.** *J Am Chem Soc* 2002, **124:**12084-12085.
32. Kokova M, Zavrel M, Tittmann K, Spiess AC, Pohl M: *Investigating the carboligase activity of thiamine diphosphate-dependent enzymes using kinetic modeling and NMR spectroscopy* Biotrans, Bern, July 5-9; 2009.
33. Gocke D, Walter L, Gauchenova E, Kolter G, Knoll M, Berthold CL, Schneider G, Pleiss J, Müller M, Pohl M: **Rational protein design of ThDP-dependent enzymes-engineering stereoselectivity.** *Chembiochem* 2008, **9:**406-412.
